# Case Report: White Colored Stool: An Early Sign of Cystic Fibrosis in Infants

**DOI:** 10.3389/fped.2021.656584

**Published:** 2021-04-14

**Authors:** Jing Guo, Rong He, Zhi-qin Mao

**Affiliations:** ^1^Department of Pediatrics, Shengjing Hospital of China Medical University, Shenyang, China; ^2^Department of Genetics, Shengjing Hospital of China Medical University, Shenyang, China

**Keywords:** cystic fibrosis, edema, hypoproteinemia, white stool, CFTR

## Abstract

A 2-month-old male infant presented with white colored stools 1 month after birth. There was no jaundice of the skin, mucous membrane, or sclera; his liver was enlarged (4 cm below the ribs), and his liver function tests showed slightly elevated total bilirubin (TB), direct bilirubin (DB), and total bile acid (TBA). An abdominal doppler ultrasound showed no signs of biliary atresia. Genetic testing revealed a *CFTR* hemizygous mutation site (c.223C>T) in exon 3 and exon 2–3 heterozygous deletion mutation. The infant's stool turned yellow after oral administration of pancreatic tablets. Finally, the infant was diagnosed with cystic fibrosis (CF). Review of literature revealed five children (including the infant in this case study) with CF who presented with white stool. All five children had anemia, four had edema and hypoproteinemia, five had changes in stool color (it was pistachio-green color in two patients, pale colored in one, acholic stool in one, and white stool in one), two had cholestasis, one infant had delayed meconium discharge, and three children had delayed growth and hepatomegaly. Two children had an abnormal sweat test, one had a F508del compound heterozygous mutation, and one had three mutation sites (C.214G>G/A, P.A72T; C.650A>A/G, P.E217G, and C.3406G>G/A, P. A1136T), which was a compound heterozygous mutation. So, CF could be included in the differential diagnosis of infants with white stool. Genetic testing could confirm an early diagnosis of CF. Pancreatic replacement therapy has been shown to be beneficial for improving the digestive function.

## Introduction

Cystic fibrosis (CF) is caused by mutations in the cystic fibrosis transmembrane transport regulator gene (*CFTR*) (1), which leads to a decrease in the secretion of chloride ions and water in epithelial cells, resulting in recurrent respiratory infections, exocrine pancreatic insufficiency, cholestasis, renal hypoplasia, and male congenital absence of vas deferens (2). The most common clinical manifestation of CF is recurrent respiratory infections; whereas, white stool is a rare initial symptom of CF (3). Here, we describe our experience with a 2-month-old male infant who presented with white stool for 1 month. He was finally diagnosed with CF. Striking white stool was the first symptom, which is different from the common clinical manifestations of CF such as, recurrent respiratory infections. We have also provided a review of the literature.

## Case Presentation

### Chief Complaints

A 2-month-old male infant was admitted to our hospital with a 1 month history of persistent white stools.

### History of Present Illness

The meconium of the child was dark green. Three days later, his stool turned yellow. After that, the stool color gradually became white stool over the next month. The test results of the infant's liver function were: albumin 16.5 g/L, total bilirubin (TB) 35.2 μmol/l, direct bilirubin (DB) 27.7 μmol/l, and total bile acid (TBA) 63.0 μmol/l. The child had taken probiotics and the symptoms did not improve.

### History of Past Illness

The patient had no significant past medical history. And the mother's pregnancy was uneventful.

### Physical Examination

At the time of admission, the infant's body weight was 3400 g (<3rd percentile) and his height was 60 cm (50–70th percentile). The physical examination revealed hepatomegaly (4 cm below the ribs with soft texture) and pitting edema of both lower limbs. No abnormality was revealed for the consciousness, cardiopulmonary examination, and nervous system examination.

### Laboratory Examinations

Routine laboratory testing upon admission showed hemoglobin was 63 g/L, packed cell volume 19.9%, normal mean-cell volume, mean cell hemoglobin concentration was at the lower end of normal, and platelets 304 × 109/L. The patient was tested negative for hepatitis A, B, and, C, syphilis, HIV, TORCH, EBV-IgM antibody, and EBV DNA; his thyroid function and blood coagulation function were within the normal range. The patient's blood glucose during admission was normal and blood and urine tandem mass spectrometry showed that the amino acid and acylcarnitine spectrum analysis were normal. Cytomegalovirus IgM test was positive. Liver function tests revealed slightly elevated levels of TB, DB, and γ-glutamyltransferase (γ-GT) (shown in Table 1).

**Table 1:** Results of liver function in children at various stages.

**Timeline**	**Age**	**ALT (U/L)**	**AST (U/L)**	**ALP (U/L)**	**TBIL (μmol/L)**	**DBIL (μmol/L)**	**TBA (μmol/L)**	**γ-GT (μmol/L)**
2020.4.20	66 days	27	63	227	35.2	27.7	63	395
2020.4.22	69 days	21	55	183	42.7	31.8	110.3	277
2020.7.29	5 months	114	210	243	57.6	46.5	105.1	198
2020.9.07	6 months	68	77	202	9.6	6.4	16	202

The cardiac ultrasound showed no abnormalities. The chest radiograph was normal. Abdominal ultrasound showed hepatosplenomegaly, liver parenchyma echoes, but the gallbladder and pancreas were normal.

### Diagnosis

**Figure 1:**
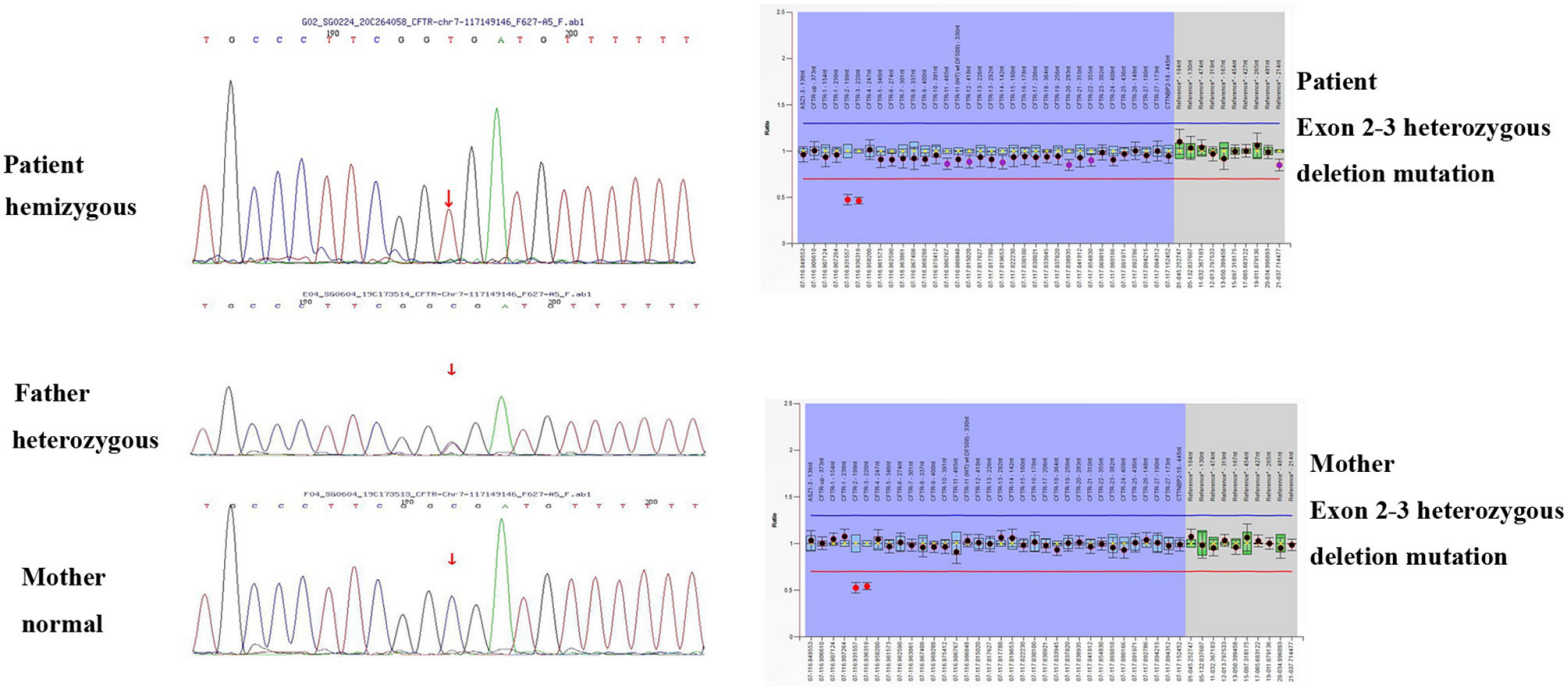
Genetic testing results of the infant and the parents.

In order to differentially diagnose whether there is biliary atresia (BA), we perfected the examination of liver, gallbladder, spleen color Doppler ultrasound, and gallbladder contraction. In order to differentially diagnose cardiogenic edema, we perfected the cardiac color Doppler ultrasound. The infant's parents refused to permit cholangiography and a liver biopsy. Due to the unclear diagnosis, we performed a genetic test. The infant's genetic analysis showed a *CFTR* hemizygous mutation site (c.223C>T) in exon 3 and exon 2–3 heterozygous deletion mutation. A genetic verification of the infant's parents was also performed; the father had a point mutation and the mother had a *CFTR* gene exon 2–3 heterozygous deletion mutation (shown in Figure 1). To further verify whether the child had pancreatic exocrine insufficiency, we tested fecal pancreas elastase 1 and the result was 0.6 μg/g, significantly lower than the normal value of 200 μg/g. Stool testing showed fat globules of 70–80/HP. These findings, combined with the manifestations of cholestasis, supported the diagnosis of CF. Because our hospitals and other testing institutions cannot perform a sweat chloride test, we did not perform it.

### Treatment and Follow-Up

**Figure 2:**
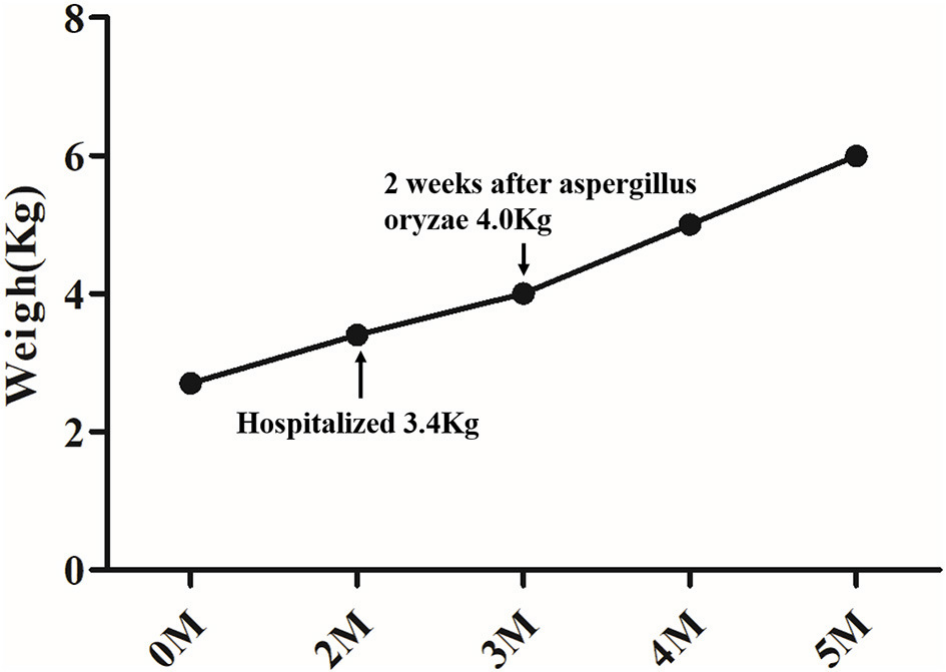
Weight change of the infant.

After hospital admission, the infant was placed on a lactose-free fortified medium-chain fatty acid milk powder feeding, oral ursodeoxycholic acid (UDCA) 30 mg/(kg.d), and supplemented with fat-soluble vitamins A, D, E, and K. The stool color remained unchanged. Fecal pancreas elastase 1 suggested that the child had insufficient pancreatic exocrine function, so aspergillus oryzae pancreatic enzymes tablets (half a tablet once, three times per day) were added 2 weeks after diagnosis. Two weeks after the oral pancreatic enzyme tablets were administered, the color of the infant's stool changed to yellow and his weight increased (shown in Figure 2).

On follow-up, liver function test was performed every 1 month and abdominal ultrasound every 2 months. The oral medication was well-tolerated by the child without adverse reactions. Two months after diagnosing the infant with CF, his TB, DB, and TBA decreased to normal levels (shown in Table 1), but the infant had a cough for 1 month, and his chest CT scan showed he had sacculus expansion of the lungs. The infant's physical examination revealed an enlarged liver (4 cm below the ribs with soft texture) in a 6-month old. Now, he is 1 year old with yellow soft stools, his body weight is 8500 g (3rd percentile), his height is 72 cm (3rd percentile). He has not had a cough for nearly 4 months, but still has an enlarged liver (2 cm below the ribs with soft texture).

### Literature Review

Using “cystic fibrosis,” “^*^ stool,” and “infant^*^” as keywords, we found three relevant articles in PubMed consisting of a total of four case reports with white stool as the first symptom; one was a domestic case report (seen in Table 2). Including this infant, there are a total of five children (three males and two females) with an average age of 2 months that have presented with white stools and diagnosed with CF. Anemia was present in all five children, edema and hypoproteinemia in four, changes in stool color in five (pistachio-green in two patients, pale colored stool one, acholic stool in one, and white stool in one patient), cholestasis in two patients, delayed meconium discharge in one, delayed growth in three, hepatomegaly in three, and splenomegaly in two infants. One child presented with respiratory symptoms at the onset.

**Table 2:** Results of literature review.

**Time**	**Author**	**Nunmber**	**Diagnosis age**	**Diagnostic basis**	**Clinical characteristics**	**Prognosis**
1997	Åke M Jakobson	2	2M/4M	Abnormal sweat test	Pistachio-green stools, anemia	Uncertain/well
2016	Li Li	1	5M	Genetic analysis	Acholic stools, cholestasis	Well
2020	Jogender Kumar	1	2M	Genetic analysis	Anemia, edema, pale colored stool	Death
2020	Jing Guo	1	2M	Genetic analysis	Acholic stool, anemia, cholestasis	Well

Two children had an abnormal sweat test: one had a F508del compound heterozygous mutation and the other child had three mutation sites (C.214G>G/A, P.A72T; C.650A>A/G, P.E217G, and C.3406G>G/A, P. A1136T), which was a compound heterozygous mutation.

The female infant (the fourth case) died because of a massive pulmonary hemorrhage. The stool changed to yellow in two infants after oral pancreatic enzymes were administered. The prognosis of the male infant (the third case) was good by UDCA and supporting treatment.

## Discussion

CF manifestation may impact multiple organ system functions (4). It most often involves the respiratory system, leading to repeated respiratory tract infections, resulting in bronchiectasis, and obstructive lung disease which eventually leads to respiratory failure. Lung disease is the most common cause of death in patients with CF. Involvement of the digestive system is manifested by insufficiency of pancreatic exocrine secretion, leading to malabsorption of fat, protein, and carbohydrates which often results in diarrhea, weight loss, steatorrhea, and malnutrition in severe cases. It can affect the liver by causing cholestasis which can progress into cirrhosis. There may also be involvement of the genitourinary system such as renal hypoplasia, male congenital absence of vas deferens, and infertility.

The incidence of CF in Slovak is 1/1,800, in Americans it is 1/20,000-1/3,000, and it is lower in Asians (5). About 70 Chinese patients have been reported in the literature, but there is no detailed epidemiological data available in China so far (6). CF is rare in China, so pediatricians have insufficient knowledge about the disease. It is easy to mistake the typical manifestations of CF, such as, repeated respiratory infections, diarrhea, and malnutrition as common children's diseases. Pneumonia, diarrhea, and malnutrition are the most common causes of mortality in children <5 years of age, children with severe CF clinical phenotypes who died in childhood may have been misdiagnosed (6). Currently, there is no newborn screening for CF in China, and it is recommended to carry out this screening.

Most of the children clinically diagnosed with CF have recurrent respiratory tract infections as their first manifestation. The key point of this case report is that white stool was the first manifestation of CF in this child. White stool in an infant usually means indigestion, pancreatic insufficiency, or biliary obstructive disease. This 2-month old infant had cholestatic hepatitis when he was admitted to the hospital. The white stools may be easily misdiagnosed as BA, but the slight increase in TB and DB did not correlate with the manifestation of biliary atresia. Hepatobiliary and spleen ultrasonography and gallbladder contraction showed no signs of typical biliary atresia such as the triangular cord sign (7). In order to further clarify the cause of acholic stool, we performed a genetic analysis. The results showed a hemizygous mutation site (c.223C>T) in exon 3 and exon 2–3 heterozygous deletion mutation, and the c.223C>T mutation which had already been reported in the literature in 2004 (8). Combined with the child's medical history, we considered the diagnosis of CF which further affects the digestion and absorption of fat and causes steatorrhea. The literature review (9–11) showed a total of four children who had white stool color as the initial manifestation of CF; suggesting that CF with white stool as the first symptom is relatively rare and easy to be missed. Most of the children had anemia, hypoproteinemia, and edema at the same time. In this case, we finally confirmed the diagnosis through genetic testing. Actually, the sweat chloride test must precede CFTR gene analysis, not only because the sweat chloride test is the gold standard for the diagnosis of CF, but also because of economic reasons. The limitation of this case report is that we did not perform a sweat chloride test. So it is important to have the possibility to perform the sweat chloride test in China or near our hospital. Therefore, a sweat chloride test and a complete genetic analysis are recommended for children who present with unexplained white stools, especially combined with anemia and hypoproteinemia; clinicians should include CF in their differential diagnosis.

Pancreatic CF often manifests as fat and protein absorption disorders caused by pancreatic exocrine insufficiency, resulting in steatorrhea, malnutrition, and delayed growth. Specific *CFTR* genotypes are significantly associated with pancreatic status, and Pancreatic Insufficiency Prevalence score is correlated with sweat chloride test (12). The gene analysis showed a hemizygous mutation site (c.223C>T) in exon 3 and exon 2–3 heterozygous deletion mutation, the mutation site (c.223C>T) has been reported to be a pathogenic mutation. So far, nearly 2,000 *CFTR* mutant genes have been discovered (13). The genetic variation differs based on the different races and geographical locations. For example, the most common mutation in European and American populations is the ΔF508 mutation (14), which is rarely reported in the Chinese population (6). Therefore, when Chinese children are suspected of having CF, whole gene sequencing is recommended to help find rare mutations, discover new mutations, and avoid missing a diagnosis of CF. CF has to be considered as a possible diagnosis for an experienced clinician.

CF cannot be cured. Pancreatic enzyme replacement therapy and symptomatic support are the main treatments. High-calorie, high-quality protein nutritional support, and supplementation of fat-soluble vitamins or trace elements are necessary. Pancreatic enzyme replacement therapy is effective (15, 16). Molecular therapy has also made great progress (17). Orkambi is a fixed-dose combination tablet containing lumacaftor and ivacaftor (LUM/IVA) which is indicated for the treatment of CF in patients 6 years and older who have a homozygous mutation of F508del (18).

In conclusion, there have been only a handful of reports citing white stools as the first symptoms of CF. CF should be considered in infants that present with white stool, especially if combined with anemia and hypoproteinemia. Genetic analysis could confirm an early diagnosis of CF. Pancreatic enzyme replacement therapy is beneficial for improving the digestive function.

## Data Availability Statement

The datasets presented in this study can be found in online repositories. The names of the repository/repositories and accession number(s) can be found below: https://www.ncbi.nlm.nih.gov/.

## Ethics Statement

Ethical review and approval was not required for the study on human participants in accordance with the local legislation and institutional requirements. Written informed consent to participate in this study was provided by the participants' legal guardian/next of kin. Written informed consent was obtained from the individual(s), and minor(s)' legal guardian/next of kin, for the publication of any potentially identifiable images or data included in this article.

## Author Contributions

JG was the patient's attending physician and he reviewed the literature and contributed to manuscript drafting. RH performed the genetic analysis. Z-qM was responsible for the revision of the manuscript for important intellectual content. All authors have approved the final version of the manuscript that is being submitted.

## Conflict of Interest

The authors declare that the research was conducted in the absence of any commercial or financial relationships that could be construed as a potential conflict of interest.
